# Murine Polyomavirus Cell Surface Receptors Activate Distinct Signaling Pathways Required for Infection

**DOI:** 10.1128/mBio.01836-16

**Published:** 2016-11-01

**Authors:** Samantha D. O’Hara, Robert L. Garcea

**Affiliations:** BioFrontiers Institute and the Department of Molecular, Cellular and Developmental Biology, University of Colorado, Boulder, Colorado, USA

## Abstract

Virus binding to the cell surface triggers an array of host responses, including activation of specific signaling pathways that facilitate steps in virus entry. Using mouse polyomavirus (MuPyV), we identified host signaling pathways activated upon virus binding to mouse embryonic fibroblasts (MEFs). Pathways activated by MuPyV included the phosphatidylinositol 3-kinase (PI3K), FAK/SRC, and mitogen-activated protein kinase (MAPK) pathways. Gangliosides and α4-integrin are required receptors for MuPyV infection. MuPyV binding to both gangliosides and the α4-integrin receptors was required for activation of the PI3K pathway; however, either receptor interaction alone was sufficient for activation of the MAPK pathway. Using small-molecule inhibitors, we confirmed that the PI3K and FAK/SRC pathways were required for MuPyV infection, while the MAPK pathway was dispensable. Mechanistically, the PI3K pathway was required for MuPyV endocytosis, while the FAK/SRC pathway enabled trafficking of MuPyV along microtubules. Thus, MuPyV interactions with specific cell surface receptors facilitate activation of signaling pathways required for virus entry and trafficking. Understanding how different viruses manipulate cell signaling pathways through interactions with host receptors could lead to the identification of new therapeutic targets for viral infection.

## IMPORTANCE

Virus binding to cell surface receptors initiates outside-in signaling that leads to virus endocytosis and subsequent virus trafficking. How different viruses manipulate cell signaling through interactions with host receptors remains unclear, and elucidation of the specific receptors and signaling pathways required for virus infection may lead to new therapeutic targets. In this study, we determined that gangliosides and α4-integrin mediate mouse polyomavirus (MuPyV) activation of host signaling pathways. Of these pathways, the PI3K and FAK/SRC pathways were required for MuPyV infection. Both the PI3K and FAK/SRC pathways have been implicated in human diseases, such as heart disease and cancer, and inhibitors directed against these pathways are currently being investigated as therapies. It is possible that these pathways play a role in human PyV infections and could be targeted to inhibit PyV infection in immunosuppressed patients.

## INTRODUCTION

Virus binding to cell surface receptors often activates signaling cascades that promote virus entry (1). Many enveloped viruses activate the phosphatidylinositol 3-kinase (PI3K) pathway to facilitate virus entry and trafficking (2). For example, hepatitis C virus (HCV) binding to CD81 and claudin-1 transiently activates the PI3K pathway to enhance virus internalization, while the Zaire Ebola virus (ZEBOV) requires PI3K activation for virus release from endosomal compartments and trafficking (3, 4). How nonenveloped viruses use signaling during virus entry is less well understood.

Polyomaviruses (PyV) are nonenveloped, double-stranded DNA viruses that rapidly induce primary host response genes (e.g., *Myc*, *Fos*, *Jun*) upon binding to cells (5–8). Primary response genes are induced by mitogenic signals at the cell surface, such as those triggering growth factor ligand binding and subsequent growth factor receptor (GFR) activation. The rapidity of PyV primary response gene induction suggests that PyV cell surface binding may activate GFRs. Many GFRs are receptor tyrosine kinases, and tyrosine kinase inhibition with genistein blocks simian virus 40 (SV40) and JC polyomavirus (JCPyV) infection, further suggesting that activation of GFRs is required for PyV infection (7, 9). However, PyVs are not known to bind GFRs directly, suggesting that other PyV receptor interactions may facilitate PyV-GFR activation.

Murine polyomavirus (MuPyV) binds to cell surface gangliosides and the α4-integrin receptor through specific sites on the VP1 capsid protein (10–12), and both receptors are required for MuPyV infection (8, 13–15). Gangliosides are sialic acid-modified glycosphingolipids that reside in the outer leaflet of the plasma membrane. Mouse embryonic fibroblasts (MEFs) lacking gangliosides cannot be infected by MuPyV, but supplementation with specific gangliosides rescues infection (8). Integrins regulate cell attachment to the extracellular matrix, cytoskeletal organization, and proliferation (16). A point mutation in the VP1 α4-integrin binding motif or knockdown of the cellular α4-integrin reduces MuPyV infection by >60% (14, 15). However, it is unclear how gangliosides or α4-integrin contribute to MuPyV infection, since MuPyV still binds to the cell surface and is internalized when these receptors are absent (8, 14, 15). Gangliosides have been shown to be required for trafficking of PyV to the endoplasmic reticulum, although the mechanism of this trafficking is unknown (17, 18). Both gangliosides and integrins are important signaling molecules that may contribute to virus activation of GFRs required for virus entry or downstream trafficking.

Both gangliosides and integrins modulate GFR activation (19–25). Gangliosides interact with GFRs in lipid rafts and can activate GFRs even in the absence of a growth factor ligand (22). Clustering of integrins through interactions with extracellular matrix proteins can also initiate and regulate signal transduction from GFRs (16, 26, 27). Fibronectin binding to α4-integrin activates transcription of primary response genes, eliciting a similar transcriptional response as induced by MuPyV binding (5, 6, 28). Interestingly, MuPyV binds to α4-integrin through the same motif as fibronectin (29), suggesting that MuPyV binding to α4-integrin could result in a similar mitogenic response. MuPyV multivalent binding to gangliosides and α4-integrin on the plasma membrane may therefore serve to cluster associated GFRs, leading to their subsequent activation. Given their important role in cell signaling, gangliosides and α4-integrin likely contribute to MuPyV-induced signaling events, downstream transcriptional changes, and infectious entry.

We describe a diverse signaling network activated immediately following MuPyV binding to the cell surface. We present evidence that interactions between VP1 and glycan receptors, gangliosides and integrins, stimulate specific signaling events required for MuPyV infection. Furthermore, we identify a subset of these signaling pathways that are critical for MuPyV entry and downstream trafficking of virus into infectious pathways.

## RESULTS

### Mouse polyomavirus activates multiple signaling pathways during virus attachment and entry.

**FIG 1:**
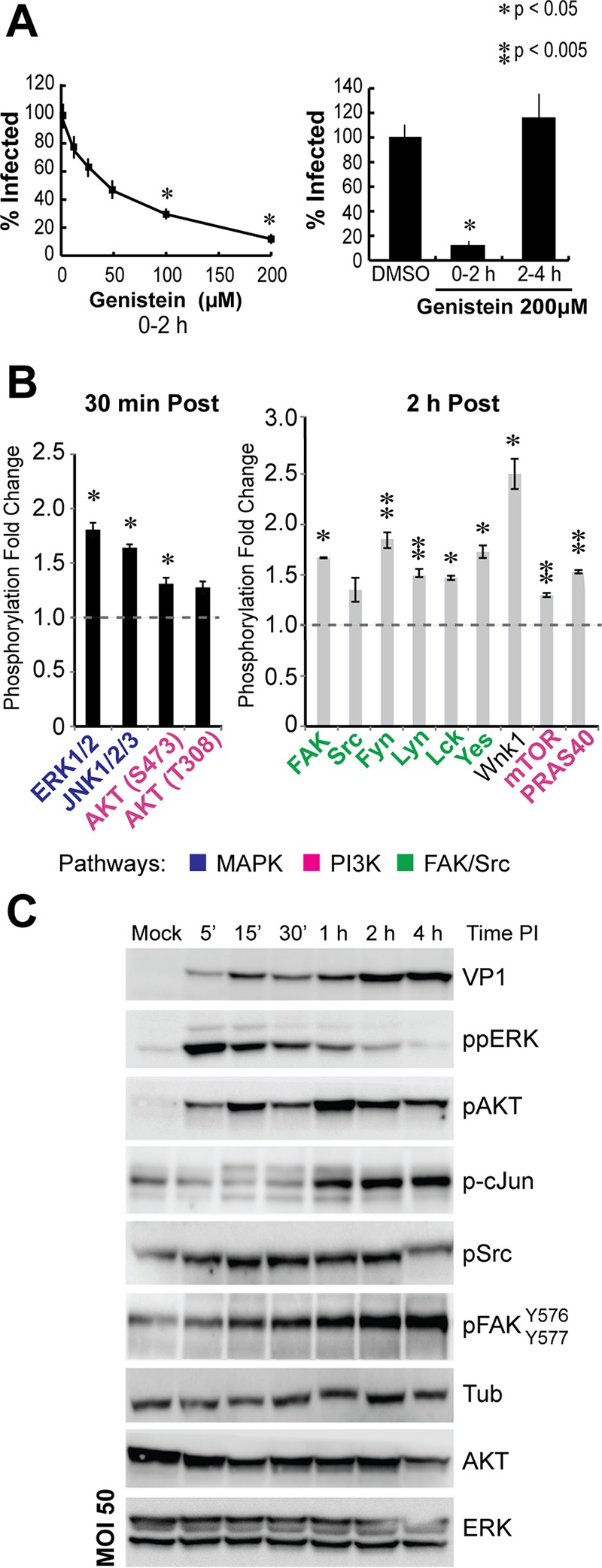
MuPyV activates required signaling pathways for infection during virus binding and entry. (A) Cells were treated with genistein during virus binding and entry (0 to 2 h) or post-virus entry (2 to 4 h). A neutralizing antibody was added after the first 2-h period. Infection was quantified at 24 h p.i. as the percentage of T-ag-positive nuclei, normalized to a DMSO control. A paired *t* test was performed (*n* = 3). (B) Phosphokinase arrays obtained at 30 min (black bars) and 2 h (gray bars) post-pseudovirus addition. Members of the MAPK, PI3K, and FAK/SRC pathways are shown on the *x* axis in blue, pink, and green, respectively. (C) Immunoblot results with MuPyV in wild-type MEFs. Multiplicity of infection (MOI), 50.

Inhibition of tyrosine kinases during virus binding to the cell surface blocks JCPyV and SV40 infection, suggesting that PyV early signaling events are essential for polyomavirus infection (7, 9). We confirmed that MuPyV infection also requires tyrosine kinase activity. MEFs were treated with the tyrosine kinase inhibitor genistein, either during virus attachment and entry (0 to 2 h), or post-virus entry (2 to 4 h). After 2 h, virus was removed and a neutralizing antibody to VP1 was added to block additional virus binding (see Fig. S1A in the supplemental material). T-antigen (T-ag) nuclear staining 24 h postinfection (p.i.) was employed to quantify the percentage of cells infected during the inhibitor treatment (see Fig. S1B). Consistent with results with JCPyV and SV40 (7, 9), genistein treatment inhibited MuPyV infection. Genistein was most effective when administered during virus entry (0 to 2 h) and blocked MuPyV infection in a dose-responsive manner (Fig. 1A; see also Fig. S1C), but it had little effect on MuPyV infectivity when the drug was added after virus entry (2 to 4 h) (Fig. 1A). These results confirmed that activation of tyrosine kinases during virus entry is required for MuPyV infection.

In order to identify specific signaling pathways activated during MuPyV binding and entry, we profiled the phosphorylation of 43 different tyrosine, threonine, and serine kinases via a phosphokinase array method (R&D Systems) after pseudovirus addition to MEFs. Pseudoviruses (PsVs) are virus-like particles that are assembled from the major (VP1) and minor (VP2/3) capsid proteins but lack an encapsidated viral genome (30). Using the phosphokinase array method, we detected four kinases phosphorylated within 30 min of pseudovirus addition (Fig. 1B), including the mitogen-activated protein (MAP) kinases extracellular signal-regulated kinases 1/2 (ERK1/2) and Jun N-terminal kinase (JNK), as well as the PI3K target AKT. Kinases phosphorylated within 2 h of pseudovirus addition included focal adhesion kinase (FAK), many of the SRC family kinases (SFKs), as well as PI3K/AKT targets, including MTOR, PRAS40, and WNK1 (Fig. 1B). We validated these array results by infecting MEFs with MuPyV and analyzing cell lysates by immunoblotting with phospho-specific antibodies. We observed phosphorylation of the earliest pathways, such as PI3K/AKT and MAPK (ERK1/2), within 5 min of virus addition (Fig. 1C). SRC family kinases were phosphorylated between 15 min and 2 h after virus addition, while both FAK and c-Jun phosphorylation increased throughout the course of infection (Fig. 1C). FAK phosphorylation was detected by 15 min, and c-Jun phosphorylation was detected 1 h after virus addition. Thus, diverse signaling networks were activated similarly by both pseudovirions and wild-type MuPyV. Network analysis of kinases detected in the array identified three major signaling pathways that were activated during virus attachment and entry—MAPK, PI3K, and FAK/SRC—and this network was significantly connected (see Fig. S1D in the supplemental material). Additionally, MuPyV infection of cells resulted in phosphorylation of the epidermal growth factor receptor (EGFR), which was previously shown to be activated during JCPyV entry (9) (see Fig. S1E).

### The ganglioside receptors GD1a and GT1a enhance PI3K activation by MuPyV.

**FIG 2:**
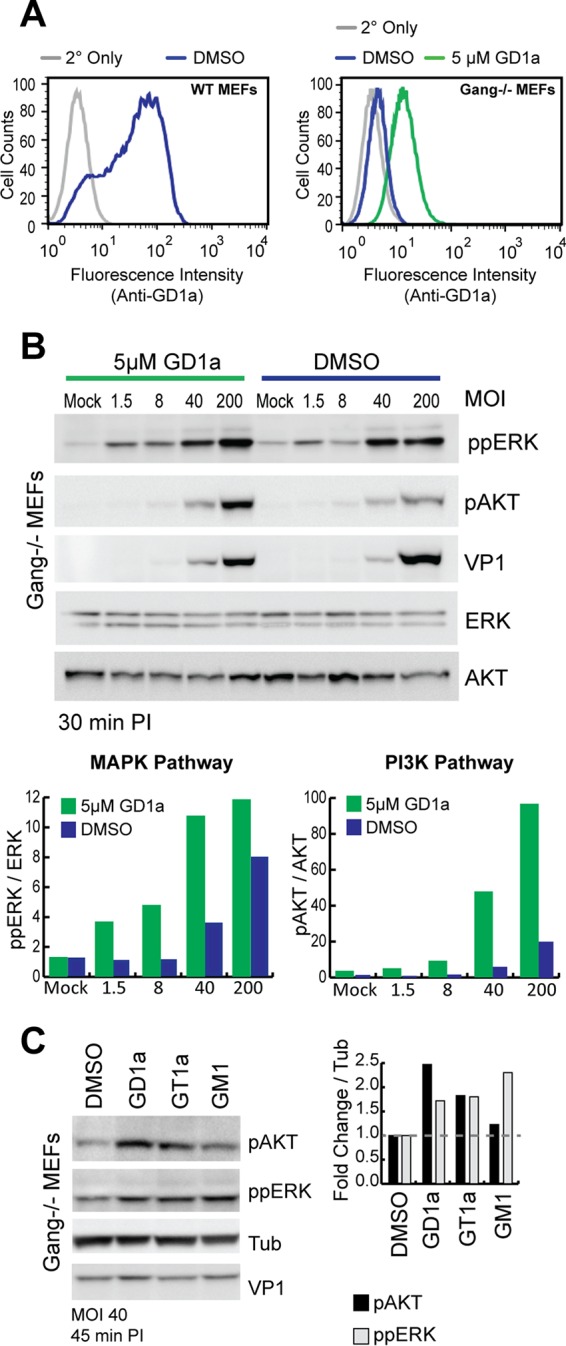
MuPyV ganglioside receptors enhance PI3K activation. (A) Flow cytometry results, displaying cell surface GD1a levels of DMSO controls for wild-type and ganglioside^−/−^ MEFs and MEFs exposed to 5 μM GD1a-supplemented ganglioside^−/−^ MEFs (green trace). Results with secondary antibody controls (2° only) are also shown. (B) Immunoblot illustrating the MuPyV dose response in DMSO or 5 µM GD1a-supplemented ganglioside^−/−^ MEFs. Bar graphs were created to quantify the integrated densities of ppERK and pAKT bands, normalized to mock treatment results. (C) Ganglioside-deficient MEFs supplemented with GD1a, GT1a, or GM1 were analyzed for signal activation after MuPyV addition (at a multiplicity of infection [MOI] of 40; 45 min p.i.). Bar graphs show integrated density results normalized to those for the mock treatment.

MuPyV does not contain known binding sites for GFRs, but MuPyV binds to gangliosides via a VP1 sialic acid binding pocket. Gangliosides are important modulators of GFR signaling (19–22), and MuPyV-ganglioside interactions could mediate MuPyV activation of GFRs and downstream signaling. Using a cell line deficient in ganglioside synthesis (ganglioside^−/−^ MEFs) that are resistant to MuPyV infection (8), we first assayed for MuPyV-induced activation of signaling pathways in the presence or absence of GD1a, a known MuPyV ganglioside receptor which restores infection of these cells (8, 13). Ganglioside^−/−^ MEFs were supplemented with 5 µM GD1a (Fig. 2A), and signaling was measured 30 min after addition of virus (Fig. 2B). GD1a supplementation alone did not alter phosphorylation of ERK or AKT, as shown by the mock sample levels (Fig. 2B). Virus addition to the dimethyl sulfoxide (DMSO) control and GD1a-supplemented ganglioside^−/−^ MEFs resulted in activation of MAPK (Fig. 2B), indicating that MAPK activation is not solely dependent on MuPyV-GD1a interactions. In contrast, AKT phosphorylation was increased 5- to 10-fold in GD1a-supplemented cells over that in the DMSO control (Fig. 2B), suggesting that GD1a mediates MuPyV activation of the PI3K pathway. Finally, to determine whether MuPyV ganglioside receptors activate specific signaling pathways versus non-MuPyV ganglioside receptors, we compared virus signal activation after supplementation with different gangliosides. The gangliosides GD1a and GT1a are known receptors for MuPyV, whereas GM1 is a receptor for SV40 PyV (13, 31). Virus addition to GD1a- and GT1a-supplemented ganglioside^−/−^ MEFs resulted in increased ERK phosphorylation compared to the DMSO control (Fig. 2C). Interestingly, virus addition to GM1-supplemented cells resulted in ERK phosphorylation above that of the DMSO control (Fig. 2C), despite the lack of interaction between VP1 and GM1, suggesting that GM1 supplementation alone may increase MAPK activation (Fig. 2B and C). In contrast, only the GD1a- or GT1a-supplemented cells showed increased phosphorylation of AKT (Fig. 2C). Taken together, these results suggest that MuPyV-specific ganglioside receptors promote activation of the PI3K/AKT pathway (Fig. 2B and C).

### α4-Integrin contributes to MuPyV signaling and infection.

In addition to specific gangliosides, MuPyV binding to α4-integrin contributes to infection (14, 15). Integrins can activate downstream signaling independently as well as through cross talk with associated GFRs; therefore, we determined whether MuPyV interactions with α4-integrin mediate MuPyV-induced signaling events. We generated two α4-integrin knockdown (α4-integrin KD) MEF cell lines that expressed ~30% of wild-type α4-integrin protein levels (see Fig. S2A in the supplemental material). Consistent with previous results (15), the α4-integrin KD MEFs showed a 60% decrease in MuPyV infection with no reduction in virus cell surface binding or ganglioside levels compared to control cells (see Fig. S2B to D). We then determined whether MuPyV-mediated signaling was altered in the α4-integrin KD MEFs. Although ERK was transiently phosphorylated between 15 and 30 min after virus addition to the α4-integrin KD MEFs, the extent of ERK activation was limited, suggesting that α4-integrin binding contributes to MuPyV activation of MAPK (see Fig. S2E). The PI3K pathway was activated in control MEFs between 15 min and 2 h after virus addition; however, in the α4-integrin KD cells, AKT phosphorylation was observed between 15 min and 30 min after virus addition. These data suggest that α4-integrin may sustain PI3K signaling after virus binding. Interestingly, c-Jun, a downstream target of many signaling pathways, showed delayed phosphorylation in the α4-integrin KD cells relative to control MEFs (see Fig. S2E), supporting a defect in MuPyV signaling. Although these data suggest a role for α4-integrin in MuPyV signal activation, it is possible that a reduction in α4-integrin levels nonspecifically alters signaling pathways, and the defect we observed was not a consequence of MuPyV binding (32). Therefore, we next tested whether pseudoviruses that are unable to bind integrins or gangliosides affect signal activation, without modifying cell surface receptor expression or cellular signaling pathways.

### VP1 binding to gangliosides and α4-integrin contributes to signal activation.

**FIG 3:**
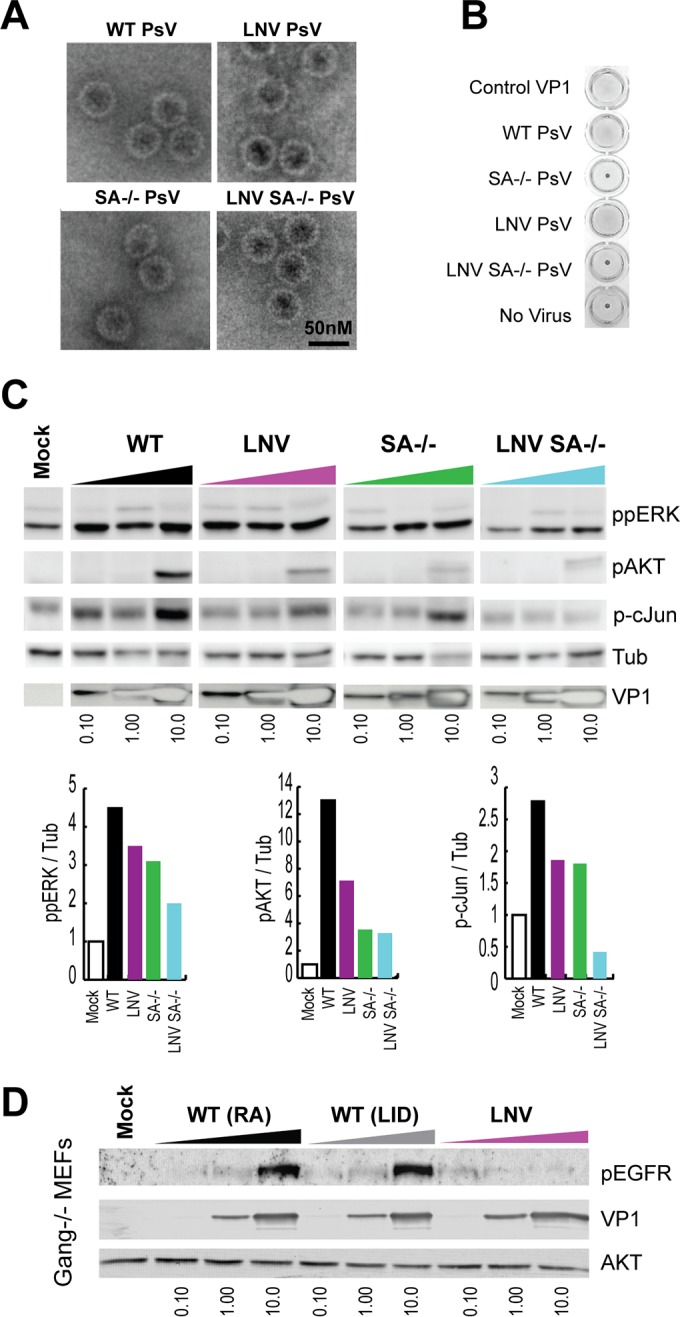
Virus binding to gangliosides and the α4-integrin receptor mediates MuPyV signal activation. (A) Electron micrograph images of wild-type and mutant PsV capsids, including the integrin binding mutant (LNV PsV), sialic acid binding mutant (SA^−/−^ PsV), and double mutant (LNV SA^−/−^ PsV). (B) Hemagglutination assay results with PsV mutants, demonstrating sialic acid binding. (C) MEFs were starved in serum-free medium followed by PsV addition. Increasing concentrations of PsV were added to cells (0.1 to 10 µg/ml). Cell lysates were collected 30 min post-PsV addition. The integrated densities of ppERK, pAKT, and pc-Jun at 10 µg/ml are shown. (D) Ganglioside-deficient MEFs were starved in serum-free medium followed by addition of wild-type (RA/LID strains) or LNV PsV. Increasing concentrations of PsV were added to cells, and cell lysates were collected 15 min post-PsV addition (0.1 to 10 µg/ml).

To confirm that gangliosides and α4-integrin binding mediate MuPyV-induced signaling, we generated mutant pseudoviruses altered by single amino acid residues in specific receptor binding sites on the VP1 capsid protein. Two residues in the sialic acid binding site of VP1 are required for sialic acid binding, H298 and R77 (33). Mutation of these amino acids (H298Q and R77Q) abrogated sialic acid binding, as shown by loss of agglutination of red blood cells (Fig. 3B, SA^−/−^). The MuPyV α4-integrin binding site is an LDV motif within VP1 that is distinct from the sialic acid binding site of VP1 (14, 15). It has been shown that changing the VP1 LDV sequence to LNV abolishes α4-integrin binding and results in a 60% decrease in MuPyV infection (14). Mutation of the integrin binding motif did not alter ganglioside (sialic acid) binding (Fig. 3B, LNV). We also generated a mutant pseudovirus lacking both ganglioside and α4-integrin binding (LNV SA^−/−^). Electron micrographs of purified wild-type and mutant pseudoviruses showed intact 50 nM capsids (Fig. 3A).

Addition of both wild-type and mutant pseudoviruses to wild-type MEFs activated the MAPK/ERK pathway (Fig. 3C). Wild-type pseudovirus induced robust AKT phosphorylation; however, induction of AKT phosphorylation by integrin (LNV^−/−^) or ganglioside (SA^−/−^) mutant pseudoviruses was greatly reduced. Furthermore, the LNV SA^−/−^ mutant pseudovirus elicited little to no AKT phosphorylation (Fig. 3C), despite high levels of virus detected by VP1 staining of whole-cell lysates (Fig. 3C). These data confirmed that both ganglioside and α4-integrin binding are required for activation of the PI3K/AKT pathway, but either interaction is sufficient for MAPK activation.

In addition to PI3K and MAPK, we observed EGFR phosphorylation after MuPyV addition to wild-type MEFs (see Fig. S1E in the supplemental material). We tested whether MuPyV activation of EGFR was ganglioside dependent, as GD1a has been shown to alter EGFR signaling (22). EGFR was activated in a dose-responsive manner in ganglioside^−/−^ MEFs by wild-type pseudoviruses (RA and LID strains), indicating that MuPyV activation of EGFR does not require ganglioside interactions (Fig. 3D). Integrin clustering can also induce EGFR activation (24). Using the integrin-deficient pseudovirus (LNV), we tested whether integrin binding contributed to EGFR activation. Addition of the LNV pseudovirus, which retains sialic acid binding, did not result in EGFR phosphorylation in ganglioside^−/−^ MEFs (Fig. 3D). These results indicate that MuPyV binding to α4-integrin can activate the EGFR.

### The PI3K and FAK/SRC pathways are required for MuPyV infection.

**FIG 4:**
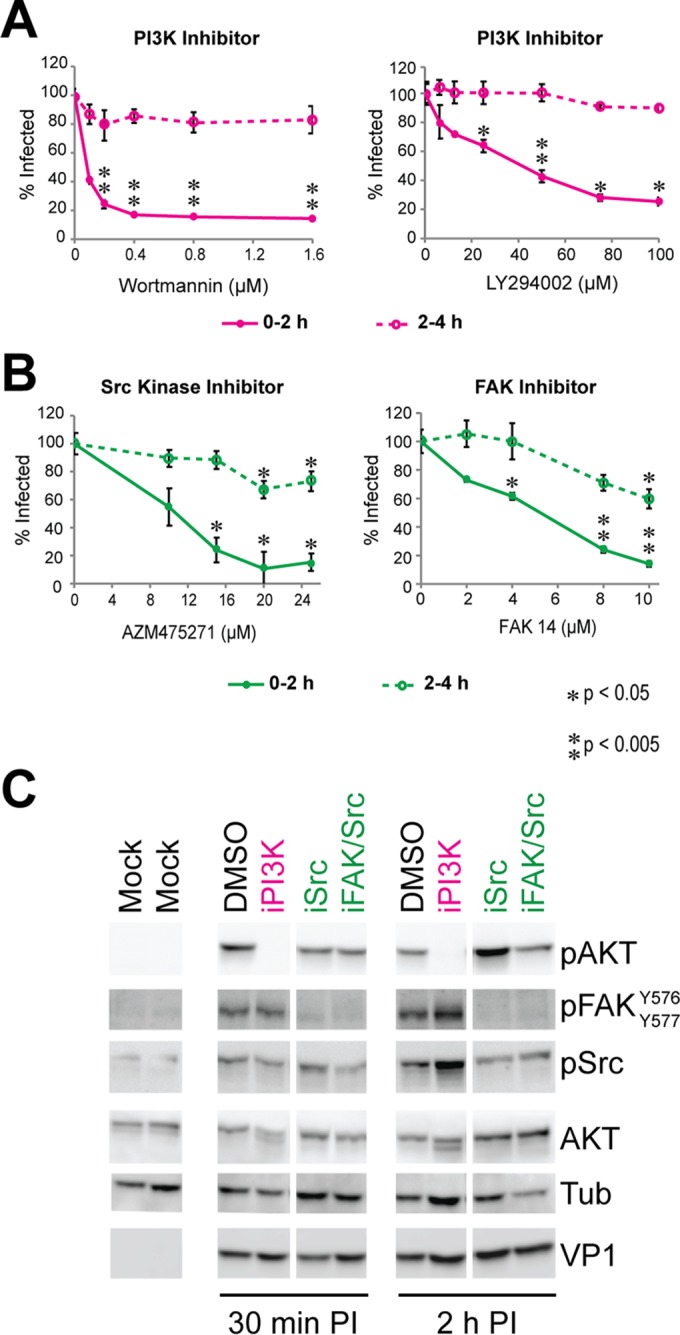
Both the PI3K and FAK/SRC pathways are required for early steps of MuPyV infection. (A) Dose-response curves for inhibitor treatments. Inhibitors were present either during virus binding (0 to 2 h; solid lines) or post-virus binding (2 to 4 h; dashed lines). The PI3K pathway was inhibited with wortmannin or LY294002. Infection was quantified at 24 h p.i. based on the percentage of T-ag-positive nuclei. A paired *t* test was performed (*n* = 3). (B) Dose-response curves for infection with the SRC kinase inhibitor AZM475271 or FAK inhibitor 14, normalized to DMSO control results. Error bars are standard errors. A paired *t* test was performed (*n* = 3). (C) Immunoblot results with cells treated with virus at a multiplicity of infection (MOI) of 50 in either the presence or absence of inhibitors for 30 min or 2 h p.i.

MuPyV cell surface binding activated the MAPK, PI3K, and FAK/SRC pathways (Fig. 1B and C). To determine which of these signaling pathways might be required for MuPyV infection, we used pathway-specific small-molecule inhibitors. Small-molecule inhibitors allowed timed inhibition of signaling specifically during early events of virus infection, without disrupting important signaling occurring during virus replication (34). Wild-type MEFs were treated with the inhibitors during virus binding and entry (0 to 2 h) or after virus entry (2 to 4 h). The PI3K target AKT was phosphorylated within 5 min of virus addition to cells, suggesting that PI3K may be important for very early steps of virus infection (Fig. 1C). Two PI3K inhibitors, wortmannin and LY294002, blocked MuPyV infection when added during virus attachment and entry, but not when added at later time points (Fig. 4A), suggesting that PI3K-mediated signaling may be important for initial steps of virus entry.

FAK and SRC family kinases were activated between 15 min and 4 h after virus addition to cells (Fig. 1B and C), suggesting that these pathways may be important for both entry and virus trafficking. We used FAK inhibitor 14 (FAK14) and a SRC family kinase inhibitor (AZM475271) to determine whether FAK/SRC activation is required for infection. Similar to the PI3K inhibitor results, inhibition of the FAK or SRC kinases during virus entry blocked infection (Fig. 4B). Inhibition of FAK/SRC post-virus entry (2 to 4 h) resulted in decreased infection, but not to the extent seen if added during virus entry (Fig. 4B). These results suggest that the FAK/SRC pathway is important for either virus entry and/or trafficking.

MuPyV binding activated the MAPK pathway (Fig. 1B and C). However, MAPK inhibition with two MEK1 inhibitors, U0126 and PD98059, had no effect on MuPyV infection (see Fig. S3A in the supplemental material), although these two inhibitors completely blocked the MAPK signal induced by MuPyV (see Fig. S3B), indicating that MuPyV activation of the MAPK pathway is not required for early events of MuPyV entry. To control for possible modulation of receptor expression by these inhibitors (35), we confirmed that ganglioside levels on the cell surfaces were at wild-type levels and there were no changes in virus binding to the cell surface (see Fig. S3C). Together, these data showed that although several signaling pathways are activated by MuPyV binding and entry, only the PI3K and FAK/SRC pathways are required for the initial steps of infection.

Many polyomaviruses activate signaling during virus entry (7, 9, 36), yet it is unclear whether the signaling pathways required for infection are conserved across species. For example, EGFR activation is required for JCPyV infection (9), and we found that the EGFR was also activated by MuPyV. However, EGFR inhibition by AG555 did not affect MuPyV infection (see Fig. S3D in the supplemental material). SV40, a monkey polyomavirus, requires caspase activation during entry (36). We tested whether caspases were functioning during MuPyV infection by using a caspase inhibitor, Z-VAD-FMK. Unlike SV40, caspase activation was not required for MuPyV infection (see Fig. S3D). Taken together, these data suggest that different PyV species utilize unique signaling pathways during virus entry.

Because the PI3K and FAK/SRC pathway are both required for infection, it is possible that these pathways are undergoing synergistic cross talk, with both contributing to a single step of infection. For example, the FAK/SRC pathway has been shown to activate PI3K (37). However, it is also possible that these pathways function independently and contribute to separate steps of infection. To determine whether cross talk occurs between the pathways during MuPyV entry, we inhibited PI3K or FAK/SRC and probed for activation of AKT, FAK, or SRC at 30 min and 2 h post-virus addition (Fig. 4C). As expected, PI3K inhibition abolished AKT phosphorylation at both 30 min and 2 h p.i. In contrast, PI3K inhibition did not decrease FAK/SRC activation. SRC kinase phosphorylates FAK at residues Y576 and Y577 (38). The SRC inhibitor blocked SRC phosphorylation of FAK Y576/577 within 30 min of infection, indicating inhibition of SRC kinase (Fig. 4C). However, inhibition of SRC did not decrease AKT phosphorylation. Simultaneous treatment with the FAK and SRC inhibitors, blocking both SRC phosphorylation of FAK and FAK phosphorylation of SRC, we observed decreased FAK and SRC phosphorylation within 30 min p.i. (Fig. 4C) without a decrease in AKT phosphorylation. Taken together, these results indicate that while the PI3K and FAK/SRC pathways are both required for infection, they are not synergistic and likely contribute to distinct steps of virus entry.

### FAK^−/−^ MEFs are resistant to MuPyV signaling and infection.

Pharmacological inhibition of FAK/SRC blocked MuPyV infection (Fig. 4B). In order to confirm that FAK is required, we tested MuPyV infection in FAK^−/−^ MEFs (39, 40). We first determined whether these cells expressed the MuPyV ganglioside receptor GD1a. Although the FAK^+/+^ MEFs showed heterogeneous expression of GD1a, with some cells expressing high levels of GD1a and others lacking the GD1a receptor (see Fig. S4A, *y* axis, in the supplemental material), the FAK^−/−^ MEFs unexpectedly displayed a complete loss of GD1a (see Fig. S4A, *y* axis), rendering them uninfectable by MuPyV. FAK^−/−^ MEFs have not been previously reported to lack cell surface gangliosides. However, even after ganglioside supplementation, confirmed by flow cytometry with a GD1a antibody (see Fig. S4B), the FAK^−/−^ MEFs remained uninfectable, suggesting that FAK is required for MuPyV infection (see Fig. S4C). Finally, we tested whether signaling pathways activated in FAK^+/+^ MEFs were activated in the absence of FAK. As expected, there was no detectable induction of SRC or AKT phosphorylation in the ganglioside-null FAK^−/−^ MEFs compared to their FAK^+/+^ controls after virus addition (see Fig. S4D). However, there were elevated levels of phosphorylated SRC in the FAK^−/−^ MEFs, as previously reported (40), although this activation was insufficient to restore infection of these cells. These results further support the conclusion that FAK is critical for virus-induced signaling events and infection.

### PI3K is important for early steps in virus entry.

**FIG 5:**
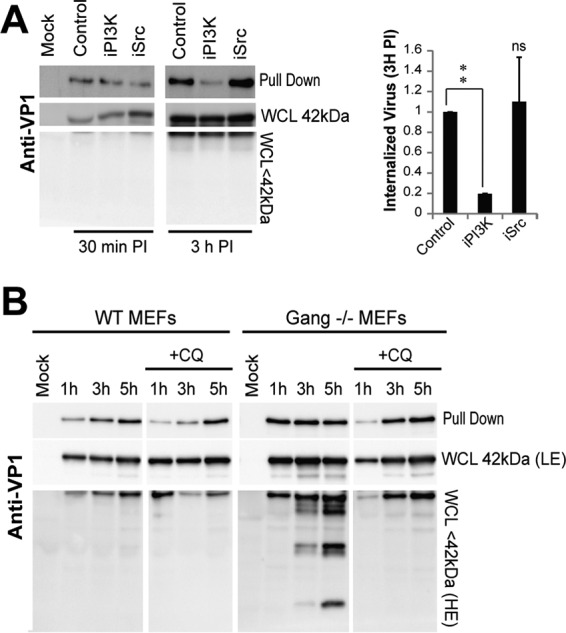
PI3K activation is required for virus internalization. (A) Internalization assays in wild-type MEFs with or without inhibitor treatment. The virus present in the WCL and observed in the streptavidin pulldown assay were detected by immunoblotting with anti-VP1 at each time point. The bar graph displays the average internalization of 2 biological replicates; error bars show standard errors. A paired *t* test was performed. **, *P* < 0.005; n.s., not significant. (B) Internalization assays in wild-type and ganglioside^−/−^ MEFs with or without the lysosomal degradation inhibitor 100 µM CQ. The virus present in the WCL and streptavidin pulldowns was detected by immunoblotting with anti-VP1 at each time point. WCL chemiluminescence low-exposure (LE) and high-exposure (HE) results are shown.

We next sought to understand how the PI3K and FAK/SRC pathways contribute to early steps in MuPyV infection. Specifically, we determined whether signal inhibition caused defects in virus endocytosis. In order to measure virus internalization, virus was covalently linked to a disulfide-biotin tag and added to cells for 30 min or 3 h at 37°C. Cells were washed with Tris-2-carboxyethylphosphine (TCEP), a non-cell-permeable reducing agent that removes biotin from virus on the cell surface while internalized virus retains the biotin tag. Whole-cell lysates (WCL) were collected and incubated with streptavidin-coated beads. The virus bound to the streptavidin-coated beads (pulldown) was eluted in 50 mM TCEP to isolate the internalized fraction. Whole-cell lysates (total virus) and the streptavidin pulldown product (internalized virus) were then immunoblotted with anti-VP1. As expected, we observed that the fraction of internalized virus increased from 30 min to 3 h in the DMSO control (Fig. 5A). However, the PI3K-inhibited sample showed no increase in internalized VP1 from 30 min to 3 h, indicating that PI3K inhibition likely reduced virus entry. It is also possible that the virus in the PI3K-inhibited samples was trafficked to lysosomes, leading to loss of the biotin tag and decreased pulldown; however, no increased virus degradation was detected in the WCL of the PI3K-inhibited samples (Fig. 5A), confirming an internalization defect during PI3K inhibition. The SRC-inhibited cells did not show a defect in virus internalization (Fig. 5A), indicating that FAK/SRC activation is not required for initial virus endocytosis and therefore is required for a later step in infection.

Ganglioside-deficient MEFs internalize MuPyV, although this internalization does not lead to infection (8). In the internalization assay, we measured virus endocytosis in ganglioside^−/−^ MEFs versus that with wild-type MEFs. Wild-type cells displayed increasing virus internalization (pulldown) from 1 h to 5 h post-virus addition with only one degradation band at less than 42 kDa (Fig. 5B). Ganglioside-deficient MEFs displayed high levels of virus internalization at 1 h p.i.; however, the amount of virus internalized (pulled down) did not increase from 1 h to 5 h p.i. Additionally, degradation of the virus was apparent at 3 h and 5 h post-virus addition and may have been due to lysosomal trafficking of the virus and subsequent loss of the biotin tag (Fig. 5B). When a lysosome inhibitor, chloroquine diphosphate (CQ), was added to ganglioside^−/−^ MEFs, the degradation was blocked and the amount of virus pulled down increased from 1 h to 5 h p.i. (Fig. 5B), supporting lysosomal degradation of the virus in the ganglioside^−/−^ MEFs. These results provide evidence that in the absence of gangliosides, MuPyV undergoes an alternative entry pathway that leads to increased lysosomal degradation. We also tested internalization in α4-integrin KD MEFs; however, there was no defect in virus internalization in these cells, suggesting that α4-integrin may be important for a later step in infection (see Fig. S5B in the supplemental material).

### FAK/SRC is important for steps in virus trafficking.

**FIG 6:**
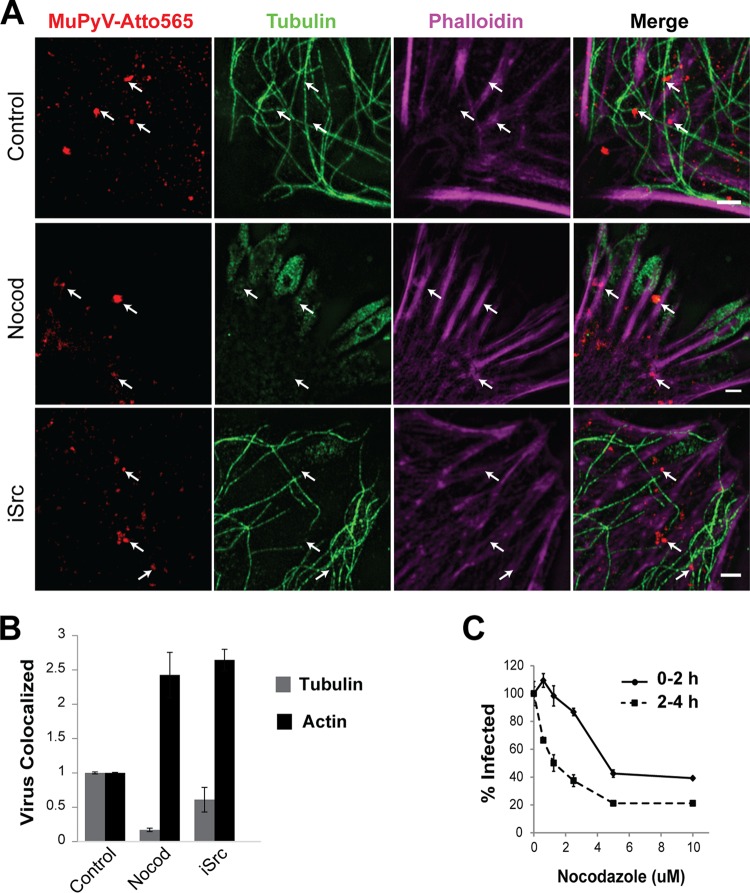
(A) SIM images of virus-treated samples 1 h postinfection. MuPyV was labeled with ATTO-565 (red), microtubules (green), and phalloidin staining actin filaments (magenta). Nocod, nocadazole. Scale bars, 2.5 µm. (B) Colocalization analysis of confocal images taken from the experiments shown in panel A. Bar graphs were used to plot the virus voxels that colocalized with tubulin voxels or actin voxels, normalized to the control. Error bars are standard errors (*n* = 2). (C) Nocodazole treatment of MEFs during virus binding and entry (0 to 2 h) or post-virus binding and entry (2 to 4 h). Infection was quantified at 24 h p.i., based on the percentage of T-ag-positive nuclei, and treatments were normalized to results with the DMSO control. Error bars are standard errors (*n* = 3).

SRC inhibition blocked infection but not MuPyV internalization, suggesting that this pathway may contribute to a subsequent step in the virus life cycle, such as virus trafficking. Microtubules have been shown to be required for MuPyV trafficking to the endoplasmic reticulum (ER) (41, 42). The microtubule polymerization antagonist nocodazole inhibited MuPyV infection when added during virus entry, and this inhibition increased when nocodazole was added during virus trafficking (Fig. 6C). In contrast, inhibition of actin polymerization increased MuPyV infection, suggesting that actin breakdown may be required for efficient virus trafficking (41) (see Fig. S3D in the supplemental material). Using confocal and superresolution structured illumination microscopy (SIM), we imaged ATTO565-labeled virus, microtubules, and actin filaments (Fig. 6A). We quantified virus association with microtubules and actin filaments 1 h post-virus addition (Fig. 6B). As expected, nocodazole treatment decreased virus association with microtubules due to microtubule depolymerization (Fig. 6B). Interestingly, nocodazole treatment increased virus association with actin at 1 h p.i. (Fig. 6B), further supporting actin depolymerization as important for MuPyV trafficking (Fig. S3D). Because the FAK/SRC pathway is known to regulate microtubule and actin dynamics (43–45), we tested virus association with microtubules and actin during FAK/SRC inhibition. We found a 40% decrease in microtubule association when cells were treated with the SRC inhibitor (Fig. 6A and B), although the microtubule network of the cell remained intact (Fig. 6A). We also observed a concurrent 2-fold increase in actin association due to SRC inhibition, indicative of virus undergoing nonproductive trafficking (Fig. 6B). These data suggest that the FAK/SRC pathway is important for virus trafficking along microtubules and that intracellular trafficking, rather than entry, is defective in the absence of FAK/SRC signaling.

## DISCUSSION

We identified a diverse signaling network activated by MuPyV cell surface binding, including the MAPK, PI3K, and FAK/SRC pathways. Activation of the PI3K and FAK/SRC pathways was required for early steps of MuPyV infection, while the MAPK pathway was not essential. PI3K activation was dependent upon VP1 interactions with both cell surface gangliosides and the α4-integrin receptor, while VP1 interactions with either gangliosides or α4-integrin were sufficient to activate the MAPK pathway. Finally, we defined the contribution of each signaling pathway to early steps of infection. We found that PI3K activation was required for virus internalization, whereas the FAK/SRC pathway contributed to virus trafficking along microtubules. These results indicate that VP1 cell surface binding activates specific signaling pathways essential for early steps of MuPyV infection.

MuPyV activation of the MAPK, PI3K, and FAK/SRC pathways is likely initiated by GFRs on the cell surface, but how the virus may activate GFR signaling is unclear, given that the capsid does not contain specific GFR binding sites. A likely possibility is that MuPyV multivalent binding to gangliosides and α4-integrin facilitates activation by indirectly clustering GFRs located in cholesterol-rich microdomains of the plasma membrane. For example, previous results showing increased transcriptional responses to complete viral capsids versus capsomere subunits suggested that clustering is important for signaling (5). In support of MuPyV GFR activation, we found that the EGFR was rapidly phosphorylated upon virus addition (see Fig. S1E in the supplemental material). Cell surface gangliosides were not required for EGFR activation by virus, although loss of both integrin and ganglioside binding abrogated EGFR phosphorylation (Fig. 3D). These results demonstrated that MuPyV activation of GFRs can be mediated by VP1 interactions with other cell surface receptors, such as α4-integrin. Sialic acid binding alone was not sufficient to activate the EGFR (Fig. 3D); thus, MuPyV does not appear to bind to the EGFR through sialic acid modifications on the extracellular domain of this receptor, or this binding is not sufficient for activation (17). Finally, although general tyrosine kinase inhibition by genistein blocked MuPyV infection, inhibition of EGFR phosphorylation alone had no effect on MuPyV infection (see Fig. S3D in the supplemental material), suggesting that multiple GFRs may contribute to signaling events required for infection.

The MAPK pathway, measured by ppERK, was activated rapidly upon virus addition to cells (Fig. 1C) through either ganglioside or integrin interactions. However, loss of both interactions resulted in decreased ppERK without loss of virus binding to the cell surface (Fig. 3C). Although rapidly activated, the MAPK pathway was not required for the early steps of MuPyV infection (see Fig. S3A in the supplemental material). It has been reported that capsid binding to cells results in increased incorporation of bromodeoxyuridine into cellular DNA (5), and it is possible that MAPK and other mitogenic signaling events occurring during entry may be important for subsequent stages of infection, such as viral DNA replication.

MuPyV rapidly induces the transcription of primary response genes (*Myc*, *Fos*, and *Jun*) upon cell surface binding (5, 6). Consistent with these observations, MuPyV binding induced c-Jun phosphorylation (Fig. 1C), which is a precursor to induction of *Jun* transcription. MuPyV binding to both ganglioside and integrins led to the highest levels of c-Jun activation, and loss of either VP1 interaction reduced phosphorylation of c-Jun (Fig. 3C). These results suggest possible cooperativity between MuPyV ganglioside and integrin binding in the activation of c-Jun. We also tested inhibition of JNK kinase and found decreased infection. However, the JNK inhibitors we tested had off-target effects, and thus it is unclear whether the JNK pathway specifically impacts infection.

The PI3K pathway, measured by pAKT, was activated rapidly upon virus addition and required both ganglioside and integrin interactions. If either interaction was lost, AKT phosphorylation was greatly reduced (Fig. 3C). It is possible that the signaling threshold required for PI3K activation is attained only when both receptors are engaged, or that a specific combination of signals is necessary. Inhibition of the PI3K pathway during virus entry blocked infection by preventing virus internalization, while inhibition post-entry had no effect (Fig. 4A and 5A). Interestingly, we found that the essential MuPyV ganglioside receptors (GD1a and GT1a) increased activation of the PI3K pathway, while non-MuPyV ganglioside receptors or ganglioside^−/−^ cells alone retained only MAPK signaling (Fig. 2B and C). These data suggest that specific VP1-ganglioside interactions may induce particular signaling pathways required for productive trafficking of virus and subsequent infection. It is important to note that even when infection-related receptors are not present, the virus still binds the cell surface and is internalized (8, 13). Thus, the productive entry pathway is a subset of the possible routes engaged by the virus. In the absence of gangliosides, virus was trafficked to the lysosome, leading to its degradation (Fig. 5B), but how gangliosides mediate trafficking of the virus to nonlysosomal or productive pathways of infection is unclear (18). In the absence of gangliosides, we observed rapid endocytosis with faster kinetics than observed in wild-type MEFs (Fig. 5C). It is unclear what facilitates this rapid uptake, but we suggest that an alternative receptor may mediate this endocytosis. In wild-type MEFs, there is competition for virus binding between gangliosides and other receptors, such as Toll-like receptor 4 (TLR4) (8, 46). In ganglioside-null cells, this competition would not exist, and increased binding to alternative receptors, such as TLR4, may induce rapid endocytosis. Interestingly, inhibition of lysosomal maturation by chloroquine diphosphate reduced the rate of endocytosis in ganglioside-null cells and restored wild-type kinetics of endocytosis (Fig. 5B), indicating that alternative pathways are dependent on endosomal maturation and trafficking to the lysosome. Gangliosides have been previously implicating in virus escape from the endolysosome to the ER; however, it is unclear how these receptors mediate this trafficking event (18). MuPyV binding to gangliosides and the subsequent activation of the PI3K pathway may define the subpopulation of viruses that escape the endolysosome and are trafficked to productive pathways for infection.

The FAK/SRC pathway modulates microtubules and actin dynamics (44), and MuPyV requires intact microtubules and disrupted actin fibers for virus trafficking (Fig. 6C; see also Fig. S3D in the supplemental material) (41). MuPyV activated FAK after the MAPK/PI3K pathways, and phospho-FAK accumulated throughout virus entry (Fig. 1C). Inhibition of the FAK/SRC pathway during either virus entry or trafficking reduced MuPyV infection (Fig. 4B). FAK/SRC inhibition did not affect virus internalization (Fig. 5A), but it decreased MuPyV-microtubule association and increased MuPyV-actin association (Fig. 6B), further implicating FAK/SRC as important for MuPyV trafficking. MuPyV activation of the FAK/SRC pathway may mediate polymerization or recruitment of microtubules to sites of virus endocytosis. Further studies investigating how microtubules are recruited to the plasma membrane during infection, as well as the role of FAK/SRC in this process, could elucidate an important step in intracellular virus trafficking.

Signaling at the cell surface appears to be a critically conserved step in PyV entry, although different PyV species may utilize distinct signaling pathways for infection (5–7, 9, 36). For example, JCPyV infection of human glial cells requires activation of the EGFR and the MAPK pathway (9), whereas MuPyV also activates EGFR and MAPK but this activation is not required for infection (Fig. 3D; see also Fig. S1E and S3A in the supplemental material). SV40 also induces phosphorylation of AKT after virus binding, but unlike MuPyV, activation of PI3K does not appear to be required for SV40 infection (36). Differences in signaling between PyVs may be due to the distinct cell surface receptors found on the host cells for these viruses. Most PyVs bind specific gangliosides as primary cell attachment receptors and it is possible that ganglioside binding induces host- or cell-specific signaling pathways. Recently, the SV40 VP1-GM1 interaction has been shown to be essential for SV40-induced vacuolization (47). Thus, SV40 binding to GM1 may induce cellular signaling pathways that cause host cell vacuolization through a similar mechanism as that mediated by GD1a and GT1a activation of PI3K after MuPyV binding.

Human PyV infections, such as those caused by BK polyomavirus (BKPyV) and JCPyV, can lead to major complications in immunosuppressed patients (48). Thus, understanding the signaling pathways required for these PyV infections could lead to new therapeutics. It is possible that PyV species that use the same ganglioside receptors may have similar signaling requirements. For example, BKPyV binds GT1b, a receptor used by MuPyV (49, 50), and thus the PI3K and FAK/SRC pathways may also play a role in BKPyV infection and could be therapeutic targets.

## MATERIALS AND METHODS

### Cells: wild-type, ganglioside^−/−^, and α4-integrin knockdown MEFs.

MEFs and ganglioside KO (ganglioside^−/−^) MEFs, obtained from Thomas Benjamin at Harvard Medical School (8), were maintained in complete growth medium (10% fetal bovine serum in Dulbecco’s modified Eagle’s medium [DMEM]). FAK^+/+^ and FAK^−/−^ MEFs were purchased from ATCC (CRL-2645 and CRL-2644, respectively) and maintained in complete growth medium. The α4-integrin KD MEFs were generated in our laboratory. Lentiviruses containing shRNAs directed against α4-integrin (RefSeq accession number NM_010576) were prepared at the Functional Genomics Facility at the University of Colorado.

Additional information regarding our materials and procedures is available in Text S1 in the supplemental material.

### Viruses and pseudoviruses.

Wild-type virus was NG59RA. Prior to addition to cells, the virus was sonicated at 70 W for 1 min and incubated at 45°C for 20 min. The solution was centrifuged at 10,000 × *g* for 3 min. The virus supernatant was then dialyzed through a 100-kDa filter (Amicon Ultra URC510096) at 10,000 × *g*. The virus was then salt extracted (washed in 850 mM NaCl), resuspended in phosphate-buffered saline (PBS), and washed an additional 2 times through the 100-kDa filter to remove contaminants. Pseudoviruses were generated following a standard protocol (30) publicly available at NCI’s Center for Cancer Research website (http://home.ccr.cancer.gov/lco/production.asp).

### Gangliosides and ganglioside supplementation.

Lyophilized gangliosides were obtained from Matreya LLC (GD1a 1062 and GM1 1061) and MyBiosource (GT1a MBS663096). Gangliosides were resuspended in serum-free DMEM and supplemented into cells for 6 h at the indicated concentrations.

### Immunoblotting and antibodies.

Cells were collected in RIPA buffer containing phosphatase inhibitors (NaF and Na_3_VO_4_) and a protease inhibitor cocktail (catalog number 11836153001; Roche). Lysates were separated by 8 to 12% SDS-PAGE and transferred to a polyvinylidene difluoride membrane. Membranes were incubated with primary antibody for 16 h at 4°C (Cell Signaling antibodies anti-pERK 4695, anti-pAKT 4058, anti-AKT 9272, anti-p-cJun 3270/9164, anti-α4-integrin 8440, anti-p-EGFR 3777, anti-pFAK 3281, and anti-pSRC 6943; Abcam’s anti-pFAK 39967) or at 37°C for 1 h (for Santa Cruz Biotechnology antibodies anti-ERK sc-93 and anti-tubulin sc-8035). Immunoblots underwent chemiluminescent development and images were obtained on the Image Quant LAS400 imager. ImageJ was used to quantify the integrated density of bands.

### Confocal microscopy.

MEFs were seeded onto glass coverslips in DMEM. At the indicated times, cells were washed in PBS and fixed with 4% paraformaldehyde. Cells were permeabilized with 0.1 to 0.5% Triton X-100 and stained for T-ag (E1) (51), GD1a (MAB5606; Millipore), or VP1 (I58). Samples were then incubated with Alexa Fluor-labeled secondary antibodies. Cells were imaged on a Nikon A1R confocal microscope.

### Flow cytometry.

For flow cytometry, cells were dissociated from the plate with Versene solution at 25°C, and suspended cells were then washed in cold PBS. Samples were fixed with 0.5% paraformaldehyde (25°C for 5 min) followed by incubation with primary antibodies. Cells were processed on a CyAn ADP analyzer.

## SUPPLEMENTAL MATERIAL

Text S1  Supplemental materials and methods. Download Text S1, DOCX file, 0.02 MB

Figure S1  (A, left) Bar graph for the dose response of neutralizing I58 anti-VP1 antibody. Virus was mixed with antibody prior to addition to cells. The I58 antibody was 100% neutralizing to a dilution of 12,500. (Right) The time course of infection at the time of neutralizing antibody addition. (B) Diagram of the drug treatment experimental protocol. (C) Genistein, a tyrosine kinase inhibitor, blocked MuPyV infection in a dose-responsive manner when treatment was added during virus binding and entry (0 to 2 h). Infection was quantified at 24 h p.i. with immunofluorescence and based on the percentage of T-ag-positive nuclei, and results were normalized to those of the DMSO control. Green, T-ag; blue, 4′,6-diamidino-2-phenylindole (DAPI). (D) The prefuse force-directed network of MuPyV-activated kinases identified in the kinase array. The network was generated based on the experimental evidence of interactions; the size of the nodes corresponds to the fold change in activation found in the kinase array, and the thickness of the edges corresponds to the overall score of the interaction. Members of the MAPK pathway are shown in blue, members of the PI3K pathway are in pink, and members of the FAK/SRC pathway and SRC family kinases are shown in green. Significantly enrichment for pathway protein-protein interactions (PPI) is shown. (E) Immunoblot of pEGFR activation 30 min and 2 h post-virus addition. Download Figure S1, TIF file, 1.9 MB

Figure S2  (A)Immunoblot for α4-integrin of KD1, KD2, and control MEF lysates. The bar graph shows integrated densities of bands normalized to the control; error bars are standard errors. (B) Representative immunofluorescence images of infection. Nuclei were labeled with by 4′,6-diamidino-2-phenylindole (DAPI; blue) and T-ag (red). The bar graph shows quantification of infection at 24 h p.i. A *t* test showed signficance at *P* = 0.009 and *P* = 0.016. (C) Immunofluorescence images for GD1a (green) and DAPI (blue) stains. (D) Flow cytometry staining for cell surface VP1 30 min post-virus addition in control and α4-integrin KD1 MEFs. Geometric means of uninfected cells and at 30 min p.i. were plotted (dashed lines shown in black and red, respectively). (E) Immunoblot of MuPyV in control and α4-integrin KD1 MEFs. Download Figure S2, TIF file, 1 MB

Figure S3  (A) Dose-response curves of MAPK inhibitor treatments (U0126, PD98059). Inhibitors were present either during virus binding (0 to 2 h; solid lines) or post-virus binding (2 to 4 h; dashed lines). Results were normalized to those for the DMSO controls, and error bars are standard errors (*n* = 3). (B) Immunoblot of ppERK and p-cJun 15 min and 24 h p.i. in the presence or absence of MEK1/2 inhibitor U0126 (20 µM). ERK was phosphorylated at 15 min p.i. The MEK1/2 inhibitor U0126 blocked ERK phosphorylation when added with the virus. However, it had no effect on virus infection, as shown by T-ag staining of lysates 24 h p.i. c-Jun was phosphorylated in both DMSO and iMEK1/2 samples at 15 min and 24 h p.i. (C) MEFs were treated with virus either in the presence or absence of inhibitors for 30 min at 4°C. Cells were fixed and stained for cell surface-bound virus (anti-VP1) and the receptor GD1a (anti-GD1a). (D) Inhibition of the EGFR, caspases, Rho-GTPases, or actin polymerization (latrunculin) during virus binding and entry (0 to 2 h) or post-virus entry (0 to 4 h). Infection was quantified at 24 h p.i. as the percentage of T-ag-positive nuclei, and treatments were compared to results with the DMSO control. Error bars are standard errors (*n* = 3). Download Figure S3, TIF file, 0.5 MB

Figure S4  A) Flow cytometry data for FAK^+/+^ MEFs (black) and FAK^−/−^ MEFs (green) 30 min post-virus addition (multiplicity of infection [MOI], 50), displayed as a contour plot with GD1a levels on the *y* axis (anti-GD1a) and virus binding on the *x* axis (anti-VP1). The geometric means of the negative controls are plotted as the gray dashed line. (B) Flow cytometry data showing GD1a staining of FAK^−/−^ MEFs and GD1a-supplemented FAK^−/−^ MEFs 5 h post-ganglioside addition. (C) Infection of FAK^+/+^ MEFs, FAK^−/−^ MEFs, and FAK^−/−^ MEFs supplemented with gangliosides GD1a or GT1b. Nuclei were stained with 4′,6-diamidino-2-phenylindole (DAPI; blue) and T-ag (Red). The bar graph shows infection of T-ag-positive nuclei at 24 h p.i. (*n* = 3). The right panel shows representative slides from the infections. Also shown are infections of wild-type, ganglioside^−/−^ MEFs, and ganglioside^−/−^ MEFs supplemented with GD1a or GT1b prior to infection. These infections were carried out alongside FAK^−/−^ infections as a positive control. (C) Immunoblot of the time course of MuPyV in FAK^+/+^ and FAK^−/−^ MEFs. Download Figure S4, TIF file, 1.3 MB

Figure S5  (A**)** Confirmation of biotin-SS-MuPyV linkage, determined by SDS-PAGE and Coomassie staining after pulldown with streptavidin-coated beads. (B) Internalization assay in wild-type, ganglioside^−/−^, and α4-integrin knockdown MEFs. (C) Infectivity of ATTO-565 MuPyV at a molar ratio of 0, 10, 20, or 40. The gel shows a Typhoon scanner image with a 560 laser. Download Figure S5, TIF file, 0.6 MB
