# Supplementary material for: Murine Polyomavirus Cell Surface Receptors Activate Distinct Signaling Pathways Required for Infection
Source: mBio. 2016 Nov 1;7(6):e01836-16. doi: 10.1128/mBio.01836-16 (PMC5090042; doi:10.1128/mBio.01836-16)
Supplement: Text S1 — Supplemental materials and methods. Download [file mbo005163052s1.docx]

**Supplemental Materials and Methods:**

**Small Molecule Inhibitor Treatment:** MEFs were plated in 96-well plates at a density of 2000 cells/well in complete growth medium and grown overnight at 37°C. To test the effect of inhibitors on MuPyV entry, MEFs were starved for 6-12 h in serum free DMEM. The virus was premixed with the indicated concentration of DMSO (control) or inhibitor and added to MEFs for 2 h. The virus and inhibitor solution were then removed and the cells were washed with serum free DMEM, followed by addition of serum free DMEM supplemented with VP1 neutralizing antibody (I58) at a dilution of 1:5000. After 2 h fresh DMEM with VP1 neutralizing antibody (1:5000) was added to cells. 20 h later the media was removed and the cells were washed with cold PBS followed by PFA fixation. To test the effect of inhibitors on MPyV targeting, MEFs were starved for 6 to 12 h in serum free complete medium followed by virus addition for 2 h. The virus solution was then removed and the cells were washed with serum free DMEM, followed by addition of serum free DMEM supplemented with either DMSO (control) or inhibitor and VP1 neutralizing antibody (I58) at a dilution of 1:5000. The VP1/inhibitor solution was removed after 2 h and cells were placed in serum free DMEM with VP1 antibody at 1:5000 dilution. 20 h later the media was removed and the cells were washed with cold PBS followed by PFA fixation. Plates were fixed in 4% paraformaldehyde at 25ºC for 10 min. Cells were then permeabilized with 0.5% Triton X-100 for 15 min at 25ºC followed by blocking in 10% FBS in PBS overnight at 4°C. Plates were then stained for the viral protein T-ag (E1 antibody) followed by incubation with Alexa Fluor labeled secondary antibody (546) and Hoechst DNA dye. The plate was then washed in PBS followed by imaging on the Molecular Devices ImageXpress Micro XL High-Content Screener. To quantify infection, T-ag staining was measured per each Hoechst stained nuclei. 5 images were collected per well, and each sample contained 3 replicates per plate. The DAPI channel on each image was thresholded and nuclei were counted using ImageJ (Analyze Particles). These particles were marked as “Regions of Interest” (ROI) and then the average pixel intensity of T-ag staining was measured for each nuclei (ROI). These were then binned into T-ag positive or T-ag negative nuclei to create % infected for each sample. The % infected reported is the average of the three plated replicates.

**SIM Microscopy and Colocalization Analysis:** For Structured Illumination Microscopy cells were plated on glass slides as previously described. Labeled virus was added at a concentration of 1 μg/ mL of VP1. At indicated times slides were fixed in 4% PFA supplemented with 0.1% glutaraldehyde, followed by 0.5% TritonX-100 permeabilization. Slides were imaged on a **Nikon N-structured illumination microscope (SIM) and images were reconstructed using the Nikon N-SIM elements module.** Confocal images for co-localization analysis were obtained on a Nikon A1R laser scanning confocal, 100X oil objective. Co-localization analysis was carried out with Imaris Coloc Software. Intensity thresholds were set for the virus and tubulin/actin channels. The % of virus voxels above threshold that co-localized with tubulin or actin voxels was then calculated by the software. Over 50 cells were quantified from each slide, with two biological replicates were performed per sample.

**Kinase Arrays:** R&D Systems Proteome Profiler^TM^ Antibody Array (Human Phospho-Kinase Array Catalog # ARY003B) were performed per manufacturer’s instructions. Briefly, MEFs were plated on 10 cm dishes at 400,000 cells per dish in complete medium and grown to 80% confluence for 24 to 48 h. MEFs were then starved in serum free DMEM for 2 to 6 h followed by pseudovirus addition. Lysates were then collected at indicated times post pseudovirus addition. Protein concentration of the lysates was quantified using a BCA assay and each sample was normalized to equivalent protein concentration. Lysates were then either added to the prepared array or aliquoted and stored at -80°C until use per manufacturers recommendation. Arrays were then processed as directed by the manufacturer. Kinase arrays were quantified using the ImageQuant^TM^ TL Array Analysis Software using the PBS control as the background subtraction.

**Plasmids and Site Directed Mutagenesis to Generate Binding Mutants:** The MPyV VP1 coding plasmid pwP, VP2 coding plasmid ph2p, and the VP3 coding plasmid ph3p were obtained from Chris Buck at the Cancer Research Center. The pwP plasmid codes for the LID strain of VP1 and was mutated to the RA strain. Further mutations in the receptor binding sites were introduced into pwP using the Quick Change Site-Directed Mutagenesis System and confirmed by sequencing.

**Virus and Pseudovirus Labeling**: Purified virus and Pseudovirus were labeled with ATTO 565 NHS-ester (Sigma **72464***)* or Biotin SS NHS-ester (ApexBio A8006). Labeling was carried out according to the manufactures suggestions and for desired theoretical molar ratio: $MR=(moles of dye)/(moles of virus)$. After the labeling reaction, free dye was quenched with hydroxylamine followed by removal using a 100 kDa spin column (Millipore UFC5100BK). Biotin-SS linkage was confirmed by pull down with streptavidin coated beads followed by SDS PAGE and Coomassie stain (Fig. S5A). ATTO-565 labeling was confirmed by Typhoon gel imager and infectivity for labeled virus was determined to a theoretical MR of 40 (Fig. S5C).

**Internalization Assay:** Biotin-SS-MuPyV was added to cells at a concentration of 1 μg/mL of VP1 for 30 min or 3 h at 37°C. Cells were washed with 50mM TCEP to remove biotin from virus on the cell surface. Cells were washed with cold PBS followed by lysis in a pull down buffer (20 mM Tris-HCl pH 8, 140 mM NaCl, 1% TX-100, 1 mM EDTA, 0.05% sodium deoxycholate). The protein concentration of cell lysates was measured by BCA assay. Lysates were resuspended at a concentration of 1mg/mL prior to streptavidin pull down. 30% of the lysate was reserved as whole cell lysate, while 70% was added to streptavidin coated beads and incubated for 1 h at 25ºC. The lysate-bead solutions were then placed on a magnetic rack for 5 min and the supernatant was discarded. The beads were washed with PBS 2x followed by resuspension in 100 uL of 50 mM TCEP in PBS and incubation for 5 min at 25ºC. The TCEP-bead solutions was placed on the magnetic rack for 5 min and the supernatant was collected (Streptavidin Pull Down). WCL and pulldowns were then separated by SDS-PAGE followed by immunoblotting for VP1 and tubulin.

**Signaling Pathways Network Analysis**: Interaction mapping between activated kinase identified in the kinase arrays was generated using SPRING and visualized using Cytoscape software. Active interactions sources for SPRING were experimental evidence, textmining, and database searching with a medium confidence level (>0.4) (1). Cytoscape generated a prefuse force directed layout based on the experimental evidence of interactions (2).

**Supplemental References:**

1. **Franceschini A**, **Szklarczyk D**, **Frankild S**, **Kuhn M**, **Simonovic M**, **Roth A**, **Lin J**, **Minguez P**, **Bork P**, **Von Mering C**, **Jensen LJ**. 2013. STRING v9.1: Protein-protein interaction networks, with increased coverage and integration. Nucleic Acids Res **41**:808–815.

2. **Christmas, Rowan; Avila-Campillo, Iliana; Bolouri, Hamid; Schwikowski, Benno; Anderson, Mark; Kelley, Ryan; Landys, Nerius; Workman, Chris; Ideker, Trey; Cerami, Ethan; Sheridan, Rob; Bader, Gary D.; Sander C**. 2005. Cytoscape: a software environment for integrated models of biomolecular interaction networks. Am Assoc Cancer Res Educ B 12–16.
