# Supplementary figures and images for: Murine Polyomavirus Cell Surface Receptors Activate Distinct Signaling Pathways Required for Infection
Source: mBio. 2016 Nov 1;7(6):e01836-16. doi: 10.1128/mBio.01836-16 (PMC5090042; doi:10.1128/mBio.01836-16)

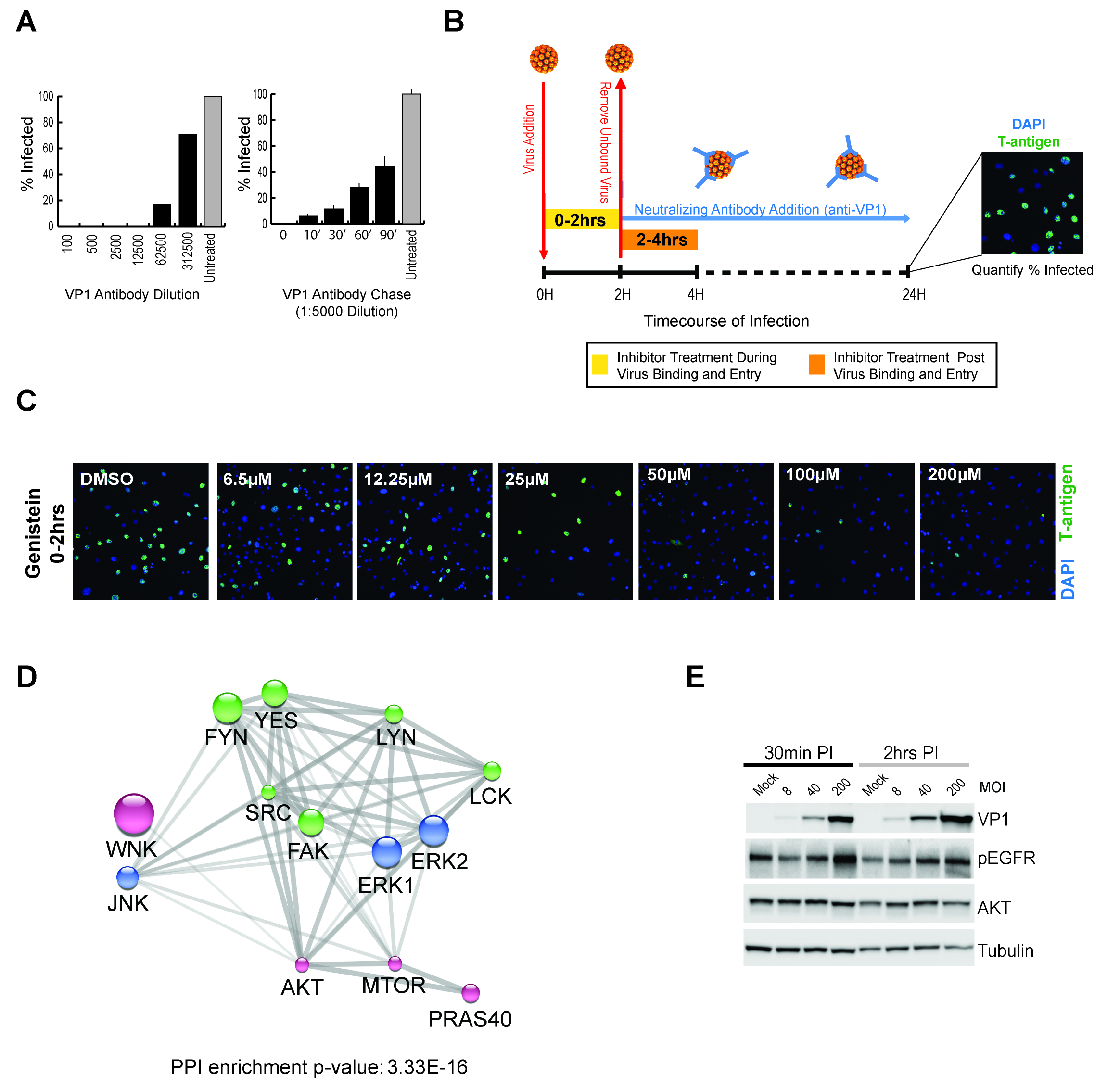

Supplement: Figure S1 — (A, left) Bar graph for the dose response of neutralizing I58 anti-VP1 antibody. Virus was mixed with antibody prior to addition to cells. The I58 antibody was 100% neutralizing to a dilution of 12,500. (Right) The time course of infection at the time of neutralizing antibody addition. (B) Diagram of the drug treatment experimental protocol. (C) Genistein, a tyrosine kinase inhibitor, blocked MuPyV infection in a dose-responsive manner when treatment was added during virus binding and entry (0 to 2 h). Infection was quantified at 24 h p.i. with immunofluorescence and based on the percentage of T-ag-positive nuclei, and results were normalized to those of the DMSO control. Green, T-ag; blue, 4′,6-diamidino-2-phenylindole (DAPI). (D) The prefuse force-directed network of MuPyV-activated kinases identified in the kinase array. The network was generated based on the experimental evidence of interactions; the size of the nodes corresponds to the fold change in activation found in the kinase array, and the thickness of the edges corresponds to the overall score of the interaction. Members of the MAPK pathway are shown in blue, members of the PI3K pathway are in pink, and members of the FAK/SRC pathway and SRC family kinases are shown in green. Significantly enrichment for pathway protein-protein interactions (PPI) is shown. (E) Immunoblot of pEGFR activation 30 min and 2 h post-virus addition. Download [file mbo005163052sf1.tif]

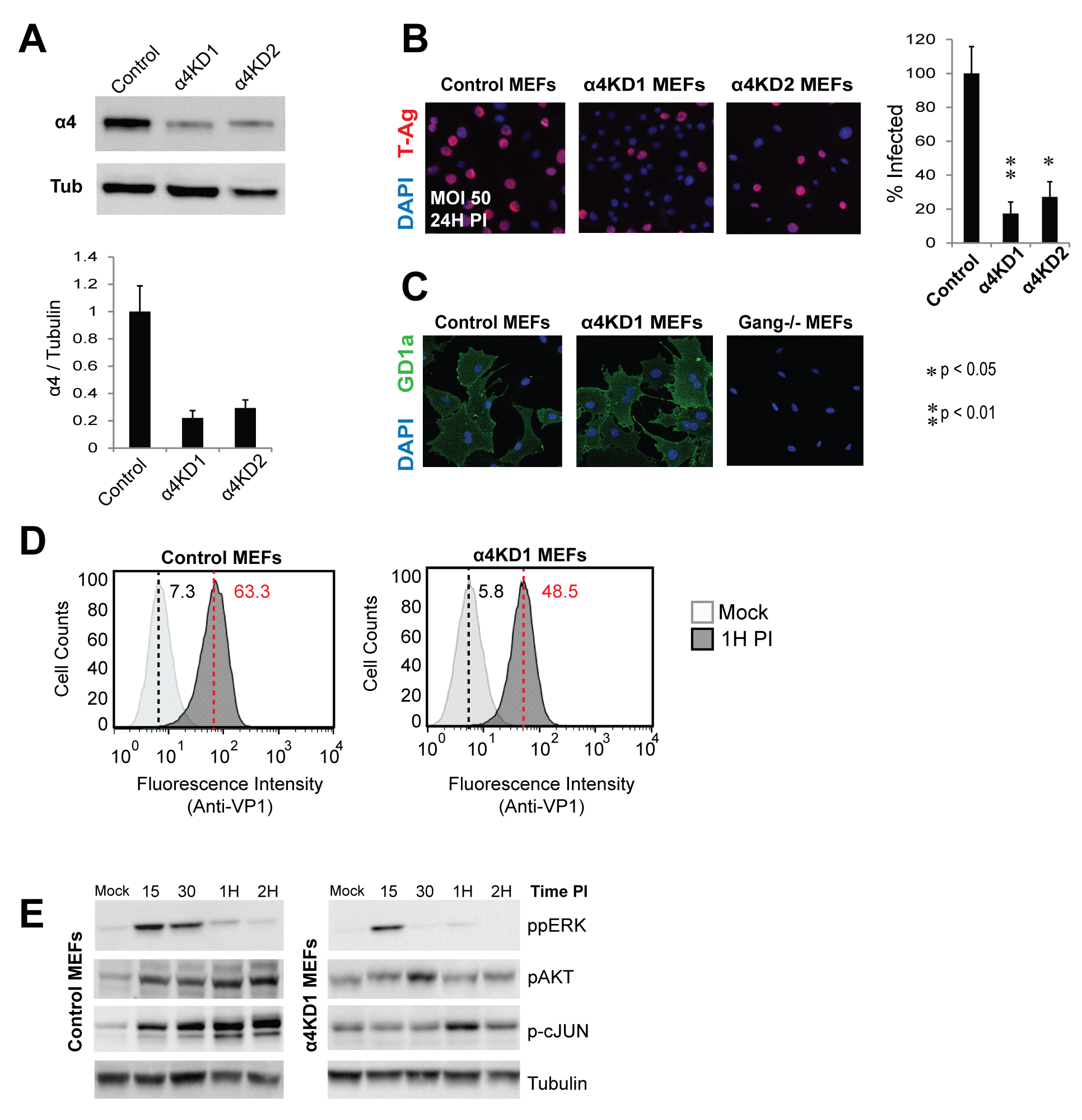

Supplement: Figure S2 — (A)Immunoblot for α4-integrin of KD1, KD2, and control MEF lysates. The bar graph shows integrated densities of bands normalized to the control; error bars are standard errors. (B) Representative immunofluorescence images of infection. Nuclei were labeled with by 4′,6-diamidino-2-phenylindole (DAPI; blue) and T-ag (red). The bar graph shows quantification of infection at 24 h p.i. A t test showed signficance at P = 0.009 and P = 0.016. (C) Immunofluorescence images for GD1a (green) and DAPI (blue) stains. (D) Flow cytometry staining for cell surface VP1 30 min post-virus addition in control and α4-integrin KD1 MEFs. Geometric means of uninfected cells and at 30 min p.i. were plotted (dashed lines shown in black and red, respectively). (E) Immunoblot of MuPyV in control and α4-integrin KD1 MEFs. Download [file mbo005163052sf2.tif]

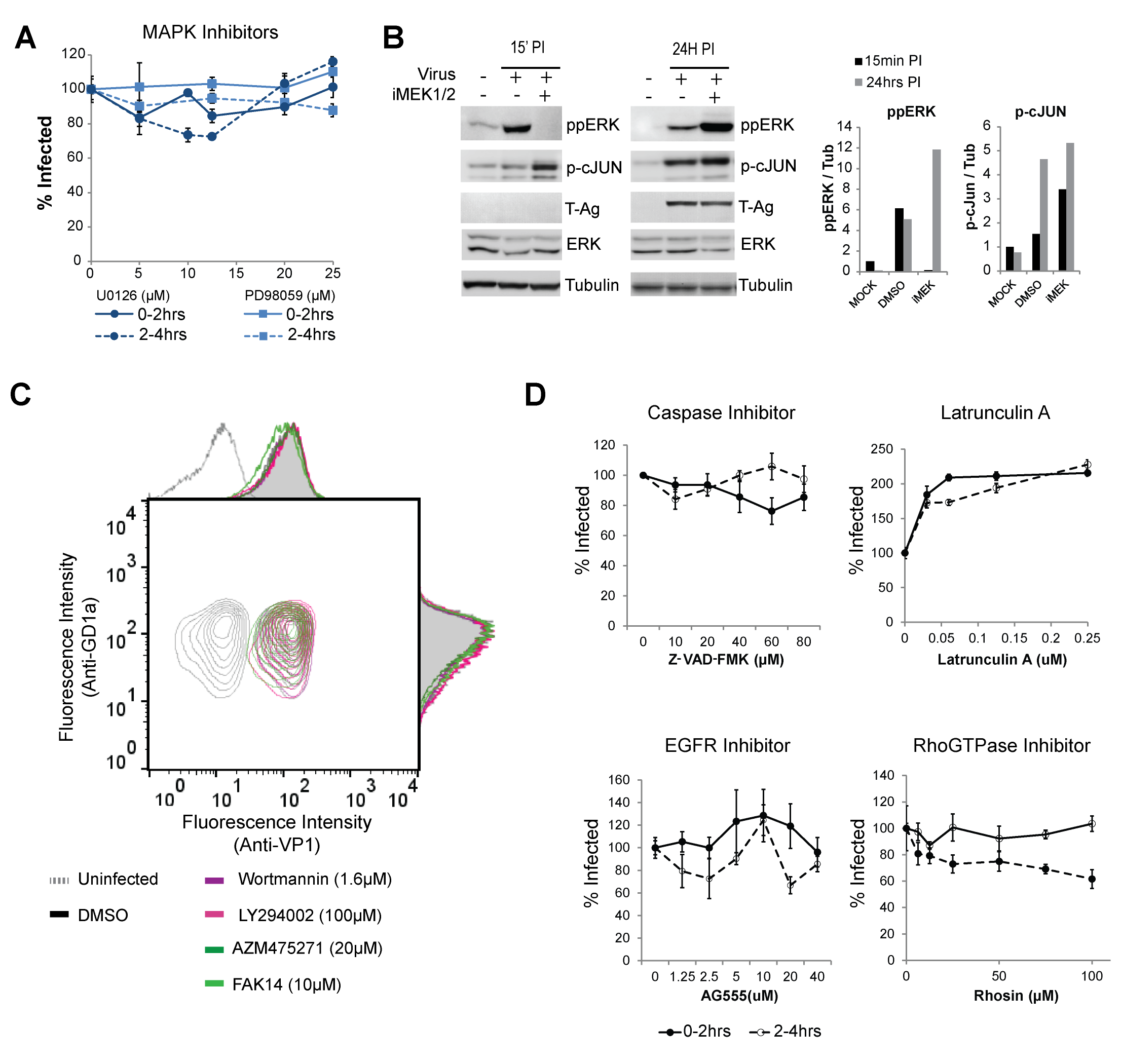

Supplement: Figure S3 — (A) Dose-response curves of MAPK inhibitor treatments (U0126, PD98059). Inhibitors were present either during virus binding (0 to 2 h; solid lines) or post-virus binding (2 to 4 h; dashed lines). Results were normalized to those for the DMSO controls, and error bars are standard errors (n = 3). (B) Immunoblot of ppERK and p-cJun 15 min and 24 h p.i. in the presence or absence of MEK1/2 inhibitor U0126 (20 µM). ERK was phosphorylated at 15 min p.i. The MEK1/2 inhibitor U0126 blocked ERK phosphorylation when added with the virus. However, it had no effect on virus infection, as shown by T-ag staining of lysates 24 h p.i. c-Jun was phosphorylated in both DMSO and iMEK1/2 samples at 15 min and 24 h p.i. (C) MEFs were treated with virus either in the presence or absence of inhibitors for 30 min at 4°C. Cells were fixed and stained for cell surface-bound virus (anti-VP1) and the receptor GD1a (anti-GD1a). (D) Inhibition of the EGFR, caspases, Rho-GTPases, or actin polymerization (latrunculin) during virus binding and entry (0 to 2 h) or post-virus entry (0 to 4 h). Infection was quantified at 24 h p.i. as the percentage of T-ag-positive nuclei, and treatments were compared to results with the DMSO control. Error bars are standard errors (n = 3). Download [file mbo005163052sf3.tif]

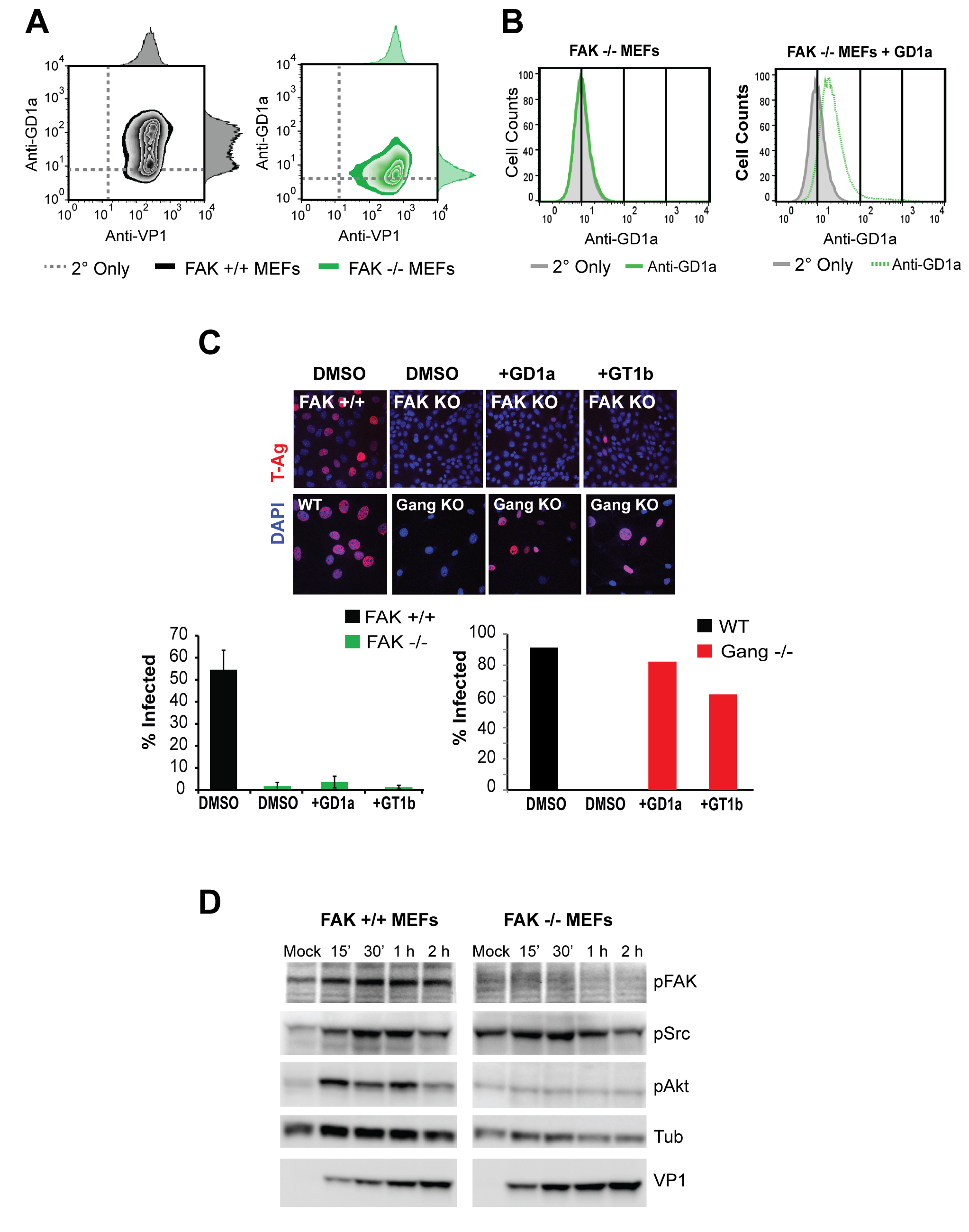

Supplement: Figure S4 — A) Flow cytometry data for FAK+/+ MEFs (black) and FAK−/− MEFs (green) 30 min post-virus addition (multiplicity of infection [MOI], 50), displayed as a contour plot with GD1a levels on the y axis (anti-GD1a) and virus binding on the x axis (anti-VP1). The geometric means of the negative controls are plotted as the gray dashed line. (B) Flow cytometry data showing GD1a staining of FAK−/− MEFs and GD1a-supplemented FAK−/− MEFs 5 h post-ganglioside addition. (C) Infection of FAK+/+ MEFs, FAK−/− MEFs, and FAK−/− MEFs supplemented with gangliosides GD1a or GT1b. Nuclei were stained with 4′,6-diamidino-2-phenylindole (DAPI; blue) and T-ag (Red). The bar graph shows infection of T-ag-positive nuclei at 24 h p.i. (n = 3). The right panel shows representative slides from the infections. Also shown are infections of wild-type, ganglioside−/− MEFs, and ganglioside−/− MEFs supplemented with GD1a or GT1b prior to infection. These infections were carried out alongside FAK−/− infections as a positive control. (C) Immunoblot of the time course of MuPyV in FAK+/+ and FAK−/− MEFs. Download [file mbo005163052sf4.tif]

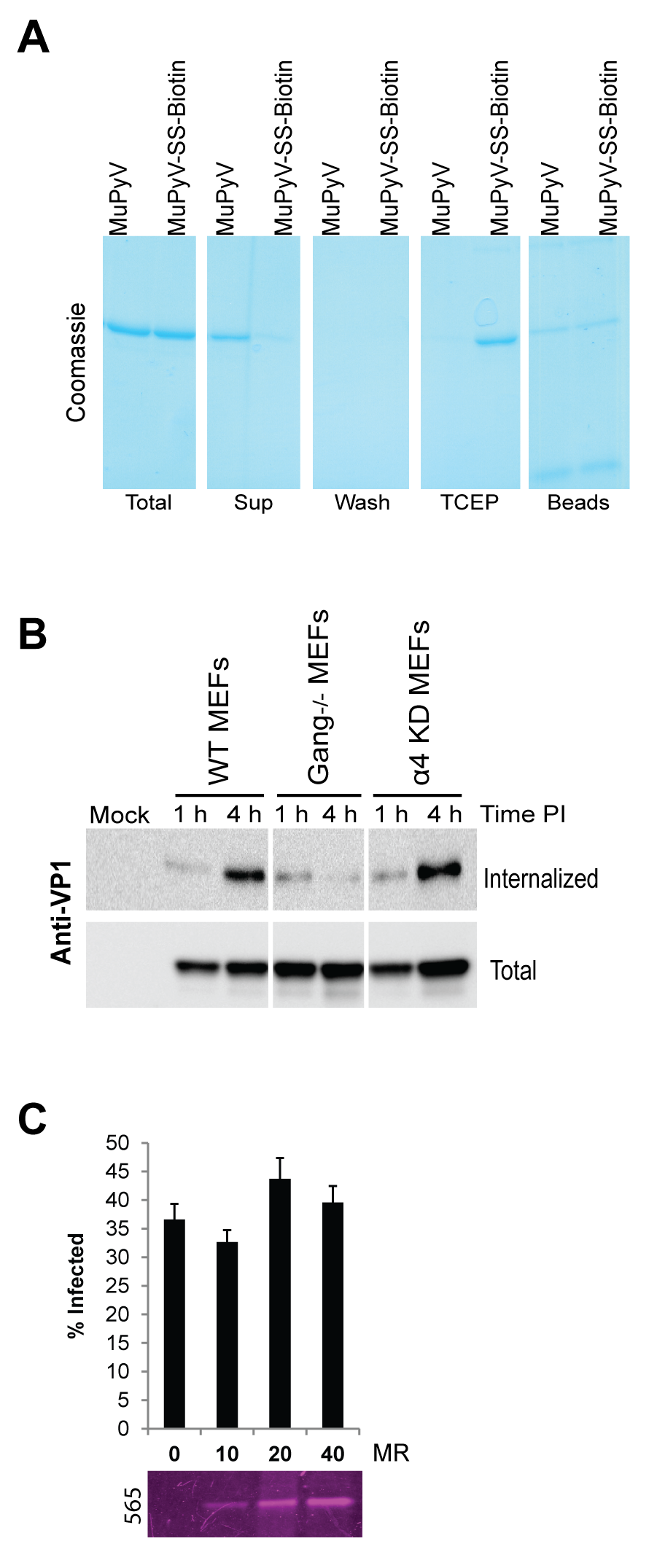

Supplement: Figure S5 — (A) Confirmation of biotin-SS-MuPyV linkage, determined by SDS-PAGE and Coomassie staining after pulldown with streptavidin-coated beads. (B) Internalization assay in wild-type, ganglioside−/−, and α4-integrin knockdown MEFs. (C) Infectivity of ATTO-565 MuPyV at a molar ratio of 0, 10, 20, or 40. The gel shows a Typhoon scanner image with a 560 laser. Download [file mbo005163052sf5.tif]
